# Caloric and Lipid Profiles in the Spanish Population of North Africa

**DOI:** 10.3390/foods11081140

**Published:** 2022-04-14

**Authors:** Miriam Mohatar-Barba, María López-Olivares, Elisabet Fernández-Gómez, Trinidad Luque-Vara, Marta Linares-Manrique, Carmen Enrique-Mirón

**Affiliations:** 1 Department of Nursing, Faculty of Health Sciences, Melilla Campus, University of Granada, Calle Santander s/n, 52001 Melilla, Spain; miriamb@ugr.es (M.M.-B.); elisabetfdez@ugr.es (E.F.-G.); triluva@ugr.es (T.L.-V.); mlinar@ugr.es (M.L.-M.); 2 Department of Nutrition and Food Science, Faculty of Health Sciences, Melilla Campus, University of Granada, Calle Santander s/n, 52001 Melilla, Spain; 3 HUM-613 Research Group, Department of Inorganic Chemistry, Faculty of Health Sciences, Melilla Campus, University of Granada, Calle Santander s/n, 52001 Melilla, Spain; cenrique@ugr.es

**Keywords:** culture, eating habits, sugar, caloric profile, lipid profile, diet quality

## Abstract

This study introduces an analysis for determining factors of diet quality among the Spanish adult population in North Africa with the aim of promoting healthier eating habits to prevent the development of diabetes mellitus. It is a diagnostic, non-experimental, cross-sectional and observational study, with a descriptive correlational methodology, with 201 participants from Ceuta and Melilla. The information collection has been carried out through the 24 h diet recall. Various sociodemographic factors influencing the quality of the diet have been analyzed. People from Melilla adopt a carbohydrate-rich diet (*p* = 0.004), whereas people from Ceuta have a lipid-rich diet (*p* = 0.002), particularly a high- monounsaturated-fat diet (*p* = 0.007). Muslims consume more sugar (*p* = 0.001) compared with Christians. Those working consume less carbohydrates (*p* = 0.13) than those not working. The latter consuming more fats (*p* = 0.39), and those with a higher education level show higher consumption of proteins (*p* = 0.001). The results of this study suggest that diet quality, in general, does not follow healthy recommendations established for the Spanish population, where the sugar consumption-diabetes relationship justifies the need for further research on Muslim population.

## 1. Introduction

Noncommunicable diseases (NCDs) are one of the major epidemics of the XXI century, including cardiovascular diseases (myocardial infarction and cerebrovascular accidents), diabetes mellitus, cancer, and respiratory diseases such as the chronic obstructive pulmonary disease and asthma [1]. In 2019, NCDs represented 74.4% of deaths worldwide, and together with overweight and obesity, they increase every year due to a plurality of risk factors including, without limitation, bad eating habits [2,3].

Bad eating habits are more and more frequent among global population and are characterised by a lack of fruits and vegetables and high consumption of saturated fats and foods high in added sugars [4]. Likewise, such nutritional imbalances are closely related to the appearance of obesity and, in particular, to type 2 diabetes mellitus (DM2) among the NCDs [5].

In Spain, according to the latest National Health Survey conducted in 2017, adult population, aged 25–60 years, showed a prevalence of obesity of 14.5%, that is, one in two adults’ weight was higher than recommended. Obesity is more frequent in women (17.5%) than in men (13.2%), incidence increases with advancing age, reaching 21.6% and 33.9% in men and women over 55 years old, respectively [6].

According to the National Statistics Institute (INE) and the WHO, diabetes is a common disease in Spain which affects 41,924 people, around 10% of population, and causes around 9300 deaths a year, being one of the main causes of death globally, involving total health expenses of 11%. Moreover, the prevalence of diabetes is increasing worldwide and studies indicate that there will be over 642 million people with diabetes by 2040 [7,8].

On the other hand, pursuant to the ANIBES (Anthropometry, Intake and Energy Balance in Spain) Study, conducted among Spanish population aged 9–75 years (the autonomous cities of Ceuta and Melilla were excluded from said study), 41.8% of child population do not comply with the rules established by the WHO regarding sugar consumption recommendations. Said percentage increases among adolescents, in particular in women (54%), it being smaller in adults and even in those aged 65–75 years, where 10.2% of participants do not follow such recommendations [9].

Eating habits are influenced by a plurality of factors, both internal (biological, emotional, or beliefs) and external (financial, social, and cultural) [10]. Undoubtedly, culture is a determining factor as regards eating habits since frequency of intakes per day, type of food, their quantity, and even times of intakes depend on culture more than on availability of foods [10,11].

However, the fact of having an unhealthy diet influenced by a certain culture does not mean that such diet cannot be modified, an intervention on the part of political and sanitary authorities being necessary in order to provide adequate nutrition education to the entire population. Thus, society would be more focused on food selection due to the healthier features thereof [12,13].

The autonomous cities of Melilla and Ceuta show multicultural and interreligious features, the Christian and Muslim religions prevailing, which has an impact on the foods eaten by the population, with respect to both food selection and times of intake, there being different eating habits [9]. Nevertheless, such habits may adversely affect the evolution of certain diseases, such as diabetes mellitus [9,14].

In that sense, Islamic culture’s eating habits are characterised by *Halal* food, that is, food not containing pork, the main animal prohibited by the Muslim or Islamic religion, as well as typical dishes based on sugar, home-made pastries and Arabic tea, a sugar-rich beverage. Such eating habits based on unhealthy foods increase the risk of suffering from DM2 [15].

In Spain, the Muslim population represents 4% of the Spanish population, being Catalonia the area with more Muslim inhabitants, where it is around 30% of the approximately 2 million of Muslims in our country. This is followed by Andalusia (18%), Madrid (15%), and Melilla and Ceuta (7%). Regarding both autonomous cities, the Muslim population in Melilla implies more than the half of the total population (54%) and 43% in Ceuta. Additionally, although it is known that approximately 9% and 8% of the population of Melilla and Ceuta, respectively, is diabetic, the exact number of diabetic Muslims and Christians with this pathology is unknown. However, it is estimated that approximately 11.5% of the Muslim population in Spain suffers from type 2 diabetes mellitus [16,17].

Another essential feature of Islamic culture is the Ramadan month, based on daily fasting for around 14 h. In the Quran, the holy book of Islam, people who are sick, such as those with diabetes, are exempt from fasting, since it may cause a plurality of consequences that may worsen diabetes, this being the main reason why health professionals recommend not to perform such practice [18].

Despite such restrictions, most Muslims with diabetes follow the fasting voluntarily, in spite of health professionals’ disapproval due to the consequences it may have. This has been recorded in different studies, where the majority of Muslims suffering from DM2 fast for at least two weeks, showing severe conditions and hypoglycemia, compared to other months [18,19,20].

The main purpose of this study is to analyse diet quality among population in the autonomous cities of Ceuta and Melilla, especially among Christians and Muslims, as well as the factors that may have an influence on such in order to promote healthier eating habits and, therefore, prevent the development of diabetes mellitus.

## 2. Materials and Methods

### 2.1. Study Design and Participants

This is a diagnostic, non-experimental, cross-sectional and observational study, with a descriptive-correlational research methodology.

Sample selection has been performed by means of a stratified probability sampling, considering gender (men and women), religion (Muslims and Christians), and place of birth (Ceuta and Melilla). The sample is composed of 201 participants, whose mean age is 34.78 ± 11.64 years old, the minimum age being 18 and the maximum, 82. Age has been statistically classified based on the quartile distribution. The sample’s features such as gender, age, religion, level of education, occupation, and place of birth are shown in Table 1.

### 2.2. Instruments and Procedure

To assess diet quality, the 24 h diet recall suggested by Rodríguez et al., (2014) [21] has been employed. By means of a survey, participants wrote, in person, the grams of food consumed the previous day in any intake consumed throughout the day (breakfast, lunch, afternoon snack, dinner and between meals) after explaining how they should estimate such amounts after breaking the dishes eaten in different foods in order to unify their notes and thus increase the reliability of results [22].

Sociodemographic data as well as data on intakes were collected upon their written informed consent between August and September 2021.

### 2.3. Statistical Analysis

Data analysis has been performed using the statistical program SPSS 26.0 for Windows establishing the level of significance at 0.05. For the descriptive study, basic statistics have been employed according to the nature of the variables. Thus, for quantitative variables, means and median have been used as the measure of central tendency, and as the measure of dispersion, standard deviation has been used, and for categorical variables, frequencies and percentages have been utilised.

For the inferential (or correlational) analysis, non-parametric tests have been used due to the non-normality of the sample revealed by means of the Kolmogorov–Smirnov test. The association among variables has been studied by means of the Chi-squared test, by using Cramer’s V as the effect size measure; as for categorical variables, and for the comparison of numerical variables, the Mann–Whiney U test has been the statistical test used. In both cases, *p* < 0.05 (95% significance) has been deemed as statistical significance value.

To verify the degree of determination that the sociodemographic variables could have on the caloric, lipid and free sugar consumption profiles, three multiple regression models were carried out with the dichotomized independent variables. In all three cases, an Ordinary Least Squares (OLS) analysis was performed to compare the dependent variables (calorie profile, lipid profile and sugar intake) with the rest of the study variables. In all regression models, unstandardized and standardized regression coefficients (β) were calculated. The β (b) coefficients can be viewed as a measure of the effect sizes associated with the non-standardized regression coefficients.

The foods collected in the 24 h recall have been turned into nutrients through the Institute of Endocrinology and Nutrition Valladolid (IENVA)’s nutrition calculator (https://calcdieta.ienva.org/tu_menu.php, accessed on 17 November 2021), the data obtained being included in the database to determine participants’ caloric and lipid profiles.

### 2.4. Ethical Considerations

The study has followed the guidelines and ethical principles of the Declaration of Helsinki. Participants were informed of the objective of the study; their participation was voluntary, and they accepted and signed the participant information and consent form.

## 3. Results

Table 2 below shows the mean and standard deviation values related to the intake of energy, macronutrients, fibre, and cholesterol together with the contribution to the total caloric value and Nutrition Goals for the Spanish population established by Spanish Society of Community Nutrition (SENC) for a daily intake [23]. The average consumption of energy is 2068.39 kcal/day and of sugar, 78.52 g/day, out of which 32.21 g/day (41%) correspond to added sugars. With respect to the caloric profile, proteins contribute 17% of the energy consumed, 41% corresponds to carbohydrates (15% in the form of simple sugar) and the remaining 42% is provided by lipids. As regards the lipid profile, the contribution to energy by saturated fats (SFs) is 13%, monounsaturated fats (MUFs) contribute 18% and polyunsaturated fats (PUFs), 8%.

Table 3 shows how the caloric profile is distributed within established limits. In this sense, we may note how more than half of participants (70.1%) consume over 15% in relation to proteins and how 75.6% tend to consume more fat than recommended (over 35%). With respect to carbohydrates, only 10.9% follow recommendations (between 50–55%), most of participants (80.6%) showing a low-carb diet with a consumption inferior to 50%. In relation to added sugars, it is worth noting that 37.8% of participants tend to consume such sugars in more than 6%.

When it comes to the contribution of different fatty acids (MUFs, PUFs, and SFs) to the lipid profile, as observed in Table 4 below, most of participants (86.6%) tend to consume over 8% of SFs, where only 5.5% of the population follow the recommended intake.

Table 5 below shows consumption of micronutrients, minerals and vitamins. With respect to minerals, it is worth noting the insufficient mean intake of calcium, iron, magnesium, zinc and potassium, whereas regarding iodine, sodium and phosphorus, the mean intake exceeds recommendations. Folic acid, vitamins C, D, E, and carotenes are consumed in smaller quantities than those recommended.

The results of the regression models carried out in relation to the caloric profile as dependent variables and the sociodemographic variables as independent variables are shown in Table 6. Carbohydrates are related to the place of birth (β = −165, *p* < 0.05) and occupation (β = 0.143, *p* < 0.05), that is, it is the people of Melilla and those with active work who consume it to a greater extent. Proteins are related to religion (β = −240, *p* < 0.05) and education (β = 0.204, *p* < 0.05), with which Christians and those with higher education have a higher consumption of this macronutrient. Finally, lipids are related to place of birth (β = 0.220, *p* < 0.05) and occupation (β= −120, *p* < 0.05), in this case, they are from Ceuta and those who do not Those with a higher intake of fat are employed.

Table 7 shows the results of two regression models carried out regarding the lipid profile and consumption of added sugars as dependent variables, and the sociodemographic variables as independent variables. Regarding the lipid profile, only a positive association with the place of birth and MUFs consumption is observed (β = 0.189, *p* < 0.05). Although both the people from Ceuta and the people from Melilla meet the recommended intake in relation to MUFs, it is the people from Ceuta who consume them to a greater extent, with no significant differences with the rest of the fatty acids (PUFs and SFs) and the sociodemographic variables. On the other hand, added sugars were positively related to religion (β = 0.234, *p* < 0.05), that is, Muslims have a higher consumption of added sugars than Christians.

## 4. Discussion

The results obtained reveal a bad diet quality according to the caloric and lipid profiles of the population studied, since they are not close to nutrition recommendations established for the Spanish population by the SENC [23].

With regard to the caloric profile, a nutritional imbalance is noted with respect to different macronutrients. In this sense, participants show a mean intake of proteins of 17% and of lipids of 42%, both above the nutrition goals established, the content of carbohydrates being of 41%, below recommendations. As regards the high consumption of lipids, they show a high level of cholesterol in intakes.

However, these results are very similar to those achieved in other studies previously conducted such as the ENIDE study [25] or the Food Consumption Panel [26]. On the one hand, according to the Food Consumption Panel, a study carried out in different autonomous cities, excluding the autonomous cities of Ceuta and Melilla, in 2008, content of carbohydrates was 41.6%, of proteins, 14.1% and of lipids, 40%. On the other hand, in the ENIDE study [25], the carbohydrate content was 41.4%, of proteins, 16% and of fats, 40.2% [26,27].

Such increase in proteins and lipids, along with a decrease in carbohydrates, has been maintained over the years. This is so reflected in ANIBES latest study conducted in 2017, where carbohydrate consumption amounted to 41.1%, of proteins, to 16.8%, and of lipids, to 38.5% [9,28].

Moreover, such an increase has also been observed in the lipid content. In this sense, the ENIDE study showed an increase in SF consumption, where population showed an intake of 12.1%, above nutrition recommendations (consumption below 8%). In the ANIBES study, consumption was of 11.7%, obtaining, in our study, an intake of 13%. None of the studies mentioned showed an increase in SFs and PUFs. However, among our participants, there was an intake of PUFs of 8%, the recommendation being below 5% [9,26,27]. It is worth noting that, in our study, people from Ceuta show a mean intake of lipid of up to 43%, far above the mean presented in the United States, such intake being of 34% [29].

As regards proteins, it is recommended that the total caloric contribution shall be of 10–15%. Nevertheless, if the goals established by the U.S. Institute of Medicine [30] are taken into account, the range is higher, being of 10–35% for adults. With respect to SFs, similar data are observed, not only in national, but also in international surveys, Australia, the United States, the Netherlands, and Denmark being the countries that show a mean intake of 13%, the same as our population, surpassed by France with a consumption of 14% [31].

The results achieved when analysing the caloric and lipid profiles with different sociodemographic variables show that people from Melilla have a carbohydrates-rich diet, above the 55% established, and that people from Ceuta tend towards a lipid-rich diet, with an intake higher than the one established for MUFs. At the same time, Christians and those with a higher education level have a higher intake of proteins, and people currently working have an intake of carbohydrates, whereas those not currently working consume more lipids than what is established. In this way, it is evident how the socioeconomic factor is present in eating habits, coinciding with several studies: those working and those with a higher education level show a healthier diet [32,33,34].

In relation to added sugars, this study notes a mean intake of 6.2%, recommendations being of less than 6%. However, consumption is much higher among Muslims (7.13%), such data being very similar to the data obtained in the latest ANIBES study, whose mean contribution of TCV was of 7.3% [9]. Besides, with reference to Benarroch’s and Pérez’s study [35], conducted with 591 secondary school students from Melilla, one third of the sample recognised consuming mass-produced pastries on a daily basis, and 41% consumed soft drinks mainly, followed by bottled juice.

Regarding micronutrients, there is a deficiency in the intake of calcium, iron, magnesium, zinc, and potassium, as well as folic acid, carotenes and vitamins C, D and E, agreeing in part. Similar results were found in adult population in the ANIBES study, where an insufficient intake of zinc, folic acid and vitamin D was evidenced [9].

It is worth mentioning that people not following a diet pursuant to the WHO’s recommendations have a higher risk of suffering from overweight, obesity, type 2 diabetes mellitus, high blood pressure, and cardiovascular diseases, as well higher cognitive impairment, higher probability of developing dementia, kidney diseases and/or lower bone density and even cancer [29,36,37,38,39,40,41,42,43,44,45]. Therefore, the need to reinforce nutrition education among population by political, education and health authorities is evident in order to limit or reduce the consumption of ultra-processed food [5].

Considering the high incidence of Muslims with diabetes living in Spain, special care should be taken as regards Ramadan fasting, despite the fact that the Quran exempt them from doing it. Today, this may be reflected in the Hassanein et al., latest study [46], in which 94.8% of DM2 people did the Ramadan fasting for over 15 days. Although one in three of them showed complications, there was a significant increase with respect to specific recommendations for fasting in such patients.

According the Agoumi et al., study [47], a nutritional imbalance was observed during the Ramadan in 100 Muslims, with a mean contribution to TCV of proteins and lipids of 27%, and with a reduction in carbohydrates of 46%, mainly due to the high consumption during such month of foods such as meat, eggs, and typical fat-rich dishes. In contrast, Guerrero et al., study [48] on Muslims from Ceuta did observe an increase in consumption of carbohydrates of 53%, primarily caused by a high intake of sweets and home-made pastries consumed during such festival.

The results of this study should be interpreted taking into account some limitations. Although, due to the sample size of the study, results cannot be generalised, and comparisons cannot be made with other publications, since there are no other papers related to this topic, it may be asserted that this is the first study conducted on this topic in the autonomous cities of Ceuta and Melilla.

That is why, with the results obtained in relation to bad eating habits, it would be interesting to broaden the sample in order to confirm the results achieved. In addition, more research is needed to clarify the nature of these relationships, as well as their potential health implications. In this way, it could be analysed with other variables not considered in this study, potential associations could be evaluated, and strategies could be devised for the promotion of health and healthy lifestyles.

Likewise, it is necessary to promote healthier eating habits to prevent the development of diabetes mellitus. In this sense, taking into account the high incidence of Muslims with diabetes who do the Ramadan with the consequences it may potentially have, it would be necessary to conduct further research on the impact on the Muslim diabetes patient both in Spain and in their different autonomous cities and communities. In this way, the knowledge about this topic could be increased in order to establish early solutions and promote healthy fasting in such community.

## 5. Conclusions

The results achieved in this study reveal that, in general, caloric and lipid profiles are far from healthy recommendations established for the Spanish population, with a low intake of carbohydrates, and a high intake of proteins, lipids, and monounsaturated saturated fats. In addition, a deficiency is observed in the intake of calcium, iron, magnesium, zinc, potassium, as well as folic acid, carotenes and vitamins C, D and E. On the other hand, there are some factors that may influence on said profiles such as religion, occupation, education level, and even place of birth. However, neither age nor gender showed any association at all.

As regards religion, Muslims show a lower intake of proteins, but sugar consumption is higher compared to Christians, who consume more proteins and less sugar. In relation to the education level, the higher the level, the higher the intake of proteins. With respect to occupation, those working consume less carbohydrates compared to those not currently working, who consume more fat. Finally, and with reference to the place of birth, people from Melilla show a higher intake of carbohydrates, but a lower intake of lipids (lower intake of MUFs) than people from Ceuta, who show a lower intake of carbohydrates and a higher intake of lipids.

## Figures and Tables

**Table 1:** Features of study sample (*N* = 201).

Sociodemographic Variables	*n* (%)
**Gender**	
Male	93 (46.3)
Female	108 (53.7)
**Age**	
Under 27 years	47 (23.4)
27–31 years	44 (21.9)
31–40 years	57 (28.4)
Above 40 years	53 (26.4)
**Religion**	
Christian	108 (53.7)
Muslim	93 (46.3)
**Level of education**	
With no higher education	51 (25.4)
With higher education	150 (74.6)
**Occupation**	
Currently not working	49 (24.4)
Currently working	152 (75.6)
**Place of birth**	
Melilla	105 (52.2)
Ceuta	96 (47.8)

**Table 2:** Mean values of energy, macronutrients, fibre, cholesterol (*n* = 201).

	Media ± DE	Median	Contribution to TCV (%)	NG
Energy (kcal)	2068.39 ± 635.08	1994.32	-	-
Proteins (g)	88.11 ± 28.98	36.65	17%	10–15%
HC (g)	212.82 ± 77.21	201.50	41%	50–55%
Sugar (g)	78.52 ± 40.99	71.99	15%	<10%
Added sugars (g)	32.21 ± 33.68	19.86	6.2%	<6%
Natural sugar (g)	46.31 ± 29.24	42.44	-	-
Starch (g)	133.94 ± 56.21	129.84	-	-
Total fat (g)	95.63 ± 38.61	92.31	42%	30–35%
SFs (g)	29.50 ± 16.27	26.14	13%	7–8%
MUFs (g)	43.44 ± 16.31	41.83	19%	20%
PUFs (g)	19.53 ± 76.64	12.09	8%	5%
Fibre (g)	18.31 ± 8.64 *	17.75 *	-	>14 g/1000 kcal
Cholesterol (mg/day)	436.70 ± 252.95	375.08	-	<300

HC: carbohydrates; TCV: total caloric value; NG: nutrition goals for the Spanish population [23]. * Fibre content every 1.000 kcal is 8.85 g.

**Table 3:** Contribution of macronutrients and sugars to TCV (caloric profile) (frequencies and percentage).

	Total (*n* = 201)
**Proteins**	
<10%	5 (2.5)
10–15%	55 (27.4)
>15%	141 (70.1)
**Lipids**	
<30%	13 (6.5)
30–35%	36 (17.9)
>35%	152 (75.6)
**Carbohydrates**	
<50%	166 (82.6)
50–55%	22 (10.9)
>55%	13 (6.5)
**Sugar**	
Added sugars	
<6%	125 (62.2)
≥6%	76 (37.8)

**Table 4:** Contribution of different fatty acids (MUFs, PUFs, and SFs) to TCV (lipid profile) (frequencies and percentages).

	Total (*n* = 201)
**SFs**	
Less than 7%	16 (8.0)
7–8%	11 (5.5)
More than 8%	174 (86.6)
**MUFs**	
Less than 20%	121 (60.2)
More than 20%	80 (39.8)
**PUFs**	
Less than 5%	95 (47.3)
More than 5%	106 (52.7)

**Table 5:** Mean intake of micronutrients of the sample being studied (*n* = 201) in relation to recommendations for the Spanish population.

	Media ± DE	Median	Recommendations for the Spanish Population ^1^	Intake
**Minerals**				
Calcium (mg)	785.92 ± 406.48	696.48	1200	I
Iron (mg)	15.28 ± 27.22	12.47	18	I
Iodine (μg)	238.55 ± 144.52	223.48	110	S
Magnesium (mg)	280.66 ± 101.10	262.41	330	I
Zinc (mg)	11.60 ± 19.24	9.88	15	I
Sodium (mg)	3476 ± 4308.10	3177.34	1500	S
Potasium (mg)	2854.76 ± 1000.95	2802.16	3500	I
Phosphorus (mg)	1345.16 ± 455.99	1295.03	700	S
**Vitamins**				
B1 (mg)	1.25 ± 0.79	1.09	0.8	S
B2 (mg)	1.64 ± 0.84	1.54	1.2	S
Niacin (mg)	32.90 ± 13.01	31.79	14	S
B6 (mg)	2.03 ± 0.91	1.82	1.6	S
Folic acid (μg)	228.47 ± 111.23	202.21	400	I
B12 (μg)	8.41 ± 19.81	4.88	2	S
C (mg)	116.09 ± 85.09	98.91	60	I
A (μg)	1290.77 ± 3563.73	662.53	800	S
Carotenes (μg)	2764.63 ± 2585.86	2133.01	4800	I
D (mg)	3.59 ± 5.20	2.15	15	I
E (mg)	6.45 ± 4.97	5.30	12	I

^1^ Recommended intake of micronutrients for the Spanish population [24]. Note: I, intake below recommendations; S, intake above recommendations.

**Table 6:** Relationship between caloric profile and sociodemographic variables.

Caloric Profile									
	HC		Proteíns				Lípids		
	B	SE	β	B	SE	β	B	SE	β
**Gender**	1.245	1.306	0.067	−0.257	0.614	−0.029	−0.757	1.146	−0.046
**Age**	0.025	0.063	0.032	−0.024	0.030	−0.062	−0.007	0.055	−0.009
**Religion**	0.744	1.301	0.040	−2.144 *	0.612	−0.240	1.456	1.142	0.089
**Place of birth**	−3.048 *	1.383	−0.165	−0.494	0.650	−0.055	3.581 *	1.214	0.220
**Level of education**	−0.312	1.750	−0.015	2.094 *	0.823	0.204	−1.403	1.536	−0.075
**Occupation**	3.063 *	1.543	0.143	−0.725	0.726	−0.070	−2.271 *	1.354	−0.120

HC: carbohydrates; B: non-standardized coefficient; SE: standard error; β: Standardized coefficient. * *p* < 0.05.

**Table 7:** Relationship between caloric profile and sociodemographic variables.

Lipid Profile						
	MUFs			Added Sugars		
	B	SE	β	B	SE	β
**Gender**	0.073	0.687	0.008	0.084	0.721	0.008
**Age**	0.023	0.033	0.056	−0.009	0.035	−0.021
**Religion**	−0.155	0.685	−0.016	20.383 *	0.719	0.234
**Place of birth**	10.811 *	0.728	0.189	−0.874	0.764	−0.086
**Level of education**	0.085	0.921	0.008	−0.158	0.967	−0.014
**Occupation**	−0.667	0.812	−0.060	0.046	0.852	0.004

B: non-standardized coefficient; SE: standard error; β: Standardized coefficient. * *p* < 0.05.

## Data Availability

The data presented in this study are available on request from the corresponding author.
